# A Comparative Analysis of Spiritual Care Needs Among Cancer Patients Receiving Home Care and Their Caregivers in Turkey

**DOI:** 10.1007/s10943-023-01988-0

**Published:** 2024-02-02

**Authors:** Sema Üstündağ, Yasemin Çekiç, Yasemin Kurtoğlu, Gamze Ünver

**Affiliations:** 1 Nursing Department, Faculty of Health Sciences, Kütahya Health Sciences University, Kütahya, Turkey; 2 Psychiatric Nursing Department, Ankara University, Ankara, Turkey; 3 Department of Family Medicine, Faculty of Medicine, Kütahya Health Sciences University, Kütahya, Turkey

**Keywords:** Cancer patients, Caregivers, Spiritual needs, Spirituality, Spiritual care

## Abstract

This study was conducted to determine and compare the spiritual care needs of cancer patients and their caregivers. A comparative descriptive, cross-sectional design was employed in this study. The study comprised 102 patients who were registered in the hospital’s home care unit, as well as their caregivers (total number = 204). The data were collected using a personal information form and the Spiritual Care Needs Inventory. The cancer patients had a mean age of 69.5 years, while their caregivers’ mean age was 53.1 years. According to the results, the cancer patients needed more spiritual care than their caregivers (*p* < 0.01). Patients’ spiritual care needs differed significantly by employment status (*p*  < 0.05). However, gender, educational level, and marital status did not have a significant difference in the spiritual care needs of the patients and their caregivers (*p* > 0.05). Moderately positive and significant (*p* < 0.05) correlations between patients and their caregivers were found for the total Spiritual Care Needs Inventory scores (r = 0.449), the meaning and hope subscale (r = 0.378), and the caring and respect subscale (r = 0.546). It is important to evaluate the spiritual needs of patients with cancer and their caregivers. In this evaluation, it is essential to elicit the perspectives of cancer patients and their caregivers concerning spiritual needs and religion. Effective spiritual care for patients and their caregivers can only be provided if their beliefs and priorities are taken into consideration.

## Introduction

Cancer is one of the leading causes of death worldwide (Sung et al., 2021). Patients and their families face significant challenges due to cancer, including reduced quality of life, physical and psychological health issues, and fears of pain, disability, and death. Cancer causes not only physical, social, and emotional challenges but also existential concerns about the meaning and purpose of life, independence, abandonment, and dignity, increasing the need for spiritual care (Chochinov et al., 2009; Delgado-Guay et al., 2013). The often unpredictable and sometimes erratic course of the disease leads to confrontation with spiritual questions and challenges (McClain et al., 2003; Delgado-Guay, 2014).

Spirituality refers to the pursuit of inner peace and purpose in the absence of religious or divine convictions. It pertains to the exploration of meaning and transcendence within the context ofan individual’s relationship with the self and others (Taylor & Mamier, 2005; Rego & Nunes, 2019). Spirituality is expressed through beliefs, values, traditions, emboli, faith, rituals, and practices (Puchalski et al., 2014; Balboni et al., 2022; Gijsberts et al., 2019). It is a dynamic and inherent aspect of human beings (Puchalski, 2012). The spiritual dimension is crucial for human well-being since it evokes positive emotions, such as faith, love, peace, and inner peace, particularly when confronting disease, bereavement, distress, and death (Gijsberts et al., 2019). The role of spiritual and religious coping in individuals diagnosed with advanced cancer is very important. Engaging in a spiritual connection with a higher power and actively seeking divine assistance have been found to improve patients’ quality of life, well-being, and ability to effectively cope with diseases (Ghorbani et al., 2021; O’Callaghan et al., 2020; Nissen & Hvidt, 2021; Rand et al., 2012; Salsman et al., 2015). Individuals who are confronted with life-threatening illnesses often experience contemplations ofa spiritual and religious nature. When they receive spiritual care, their spiritual well-being increases and quality of life improves (Tarakeshwar et al., 2006; Bai & Lazenby, 2015). The primary objective of care provided for these individuals is to manage symptoms, reduce pain, and offer peaceful end-of-life care while upholding the principles of dignity. Spiritual care, an integral part of holistic care, is considered an important indicator of quality of care. The provision of spiritual support by healthcare personnel is an essential component in the holistic care of cancer patients (Peppercorn et al., 2011). Spiritual care is defined as the recognition and consideration of spirituality in healthcare. It is imperative to incorporate spiritual interventions in the care plan of cancer patients. The provision of spiritual care entails the collaborative efforts of doctors, nurses, social workers, and spiritual caregivers. A spiritual history is necessary for a complete medical history. Regular spiritual assessment and education are crucial for those facing life-threatening illnesses and their families. It is essential to meet the physical, psychological, and spiritual needs of patients and involve patients’ families in the care process. The first requirement for effective spiritual care is to assess the spiritual needs of patients and their significant others (Ghorbani et al., 2021; O’Callaghan et al., 2020; Nissen & Hvidt, 2021; Rego & Nunes, 2019). This encompasses life review, hopes, and fears, quest for purpose and meaning, beliefs, acts of forgiveness, and tasks related to achieving a sense of fulfilment in life (Puchalski, 2012; Balboni et al., 2022; Rego & Nunes, 2019). Home care services should comprehensively address the various dimensions of patients and caregivers’ physical, social, mental, and spiritual needs. Being aware of patients’ spiritual care needs improves care quality. The aim of this study was to determine the spiritual care needs of patients with cancer and their primary caregivers within the context of home healthcare services.

## Methods

### Research Design

A comparative descriptive, cross-sectional design was employed in this study.

### Participants

In this study, no sample selection method was used; however, some cancer patients and their caregivers ‘were excluded due to their lack of consent for participation in the study (n = 36) or illiteracy (n = 28). As a result, the sample consisted of 102 cancer patients and their caregivers (total number of participants = 204), and the response rate was 60.7%. The patients and their caregivers were informed about the study, and their informed consent was obtained on a voluntary basis.

### Inclusion Criteria

Cancer patients aged over 18 years who were registered with a home health care service and did not have any communication problems were enrolled in the study. The caregivers were required to have provided care for a cancer patient for six months or longer, being at least18 years of age, and not having any communication difficulties. In this study, the term ‘caregiver’ was defined as the person who provided the most physical and/or emotional support throughout the patient’s illness.

#### Exclusion Criteria

Excluded from the study were patients aged under 18 years; those with limitations in reading, hearing, vision, speech, or comprehension; and those who were not registered with home health care services. For the caregiver group, individuals under the age of 18 years, those who had provided care for a cancer patient for less than six months, and those with specific disabilities were not included.

#### Data Collection Process

The research was undertaken at a university hospital in Turkey from March 15, 2022, through September15, 2022. At the beginning of the study, the patients and their caregivers provided informed consent on a voluntary basis. The researchers conducted face-to-face interviews with patients from the home care department who met the eligibility criteria and collected the necessary data for the study. The interviews had a mean duration of 15–20 min.

### Measurements

Data for the study were collected using a personal information form and the Spiritual Care Needs Inventory.

#### Personal Information Form

The researchers prepared two separate forms, one tailored for patients and the other for caregivers, in light of the literature (Ayık et al., 2021; O’Callaghan et al., 2020; Buck & McMillan, 2012).

#### Personal Information Form for Patients

This form was used to gather information regarding the sociodemographic and clinical characteristics of the patients, including age, gender, marital status, education level, occupation, socioeconomic status, employment status, and disease-related details (type and duration of the disease).

### Personal Information Form for Caregivers

This form was used to collect data concerning the caregivers’ age, gender, marital status, educational level, employment status, income, and relationship to the patient.

### Spiritual Care Needs Inventory (SCNI)

Developed by Wu et al. in 2016 this inventory evaluates the spiritual care needs of patients. The reliability and validity analyses of the Turkish version of this inventory were undertaken by İsmailoğlu et al. in 2019.This instrument consists of 21 items on patients’ potential spiritual care needs under five constructs. The items on the inventory are scored on a five-point Likert-type scale from 1 to 5 as follows: 1, ‘not at all necessary’; 2, ‘not necessary’; 3, ‘not important’; 4, ‘absolutely necessary’; and 5, ‘absolutely necessary’. The scale is divided into two subscales: ‘meaning and hope’ and ‘caring and respect’. The meaning and hope subscale score indicates an individual’s spiritual well-being in relation to oneself, nature, and the environment. The Cronbach’s alpha internal consistency coefficient was reported to be 0.93 for the meaning and hope subscale and 0.90 for the caring and respect subscale (İsmailoğlu et al., 2019). The minimum score of the scale is 21, and the maximum score is 105. An increase in the total mean score on the scale shows that the patient needs more spiritual care. In this study, the Cronbach’s alpha coefficient of the scale was calculated as 0.856.

### Ethical Considerations

This study was conducted in accordance with the principles of the Declaration of Helsinki. Approval was granted by the Non-Interventional Clinical Research Ethics Committee of Kütahya Health Sciences University (Date: 09 February 2022, Decision number: 2022/02–04). Institutional permission was also obtained. Prior to participation, the patients and their caregivers were given comprehensive information regarding the purpose and methodology of the study, and all participants provided informed consent.

### Data Analysis

The data that was collected was analysed using the SPSS for Windows, version 22.0 (IBM Corp., Armonk, NY, USA). Mean, standard deviation, range, and percentage values were used as descriptive statistics to characterise the patients and their caregivers. To evaluate variable disparities based on the spiritual needs of the participants, the study employed the independent-samples t-test, and one-way ANOVA. Correlations between the sub-dimensions of variables in cancer patients and their caregivers’ spiritual need scores were examined using Pearson correlation coefficients. Statistical significance was set at 0.05.

## Results

The mean age of the participants was 61.33 ± 16.16 years, with more than half of the participants being female (59.8%) and the majority being married (86.3%). Of the participants, 70.1% had completed primary school education, and the majority (86.3%) were unemployed. At the time of the study, the predominant diagnosis among the patients was lung cancer (14.7%), and 33.4% of caregivers had a chronic disease. According to the results of the study, a significant majority of the patients and caregivers engaged in regular religious rituals (84.3%), with the most commonly performed religious ritual being prayer (74.4%). The socio-demographic and clinical characteristics of the participants are presented in Table 1.

**Table 1:** Socio-demographic characteristics of cancer patients and caregivers (n = 204)

Characteristics	Patients number (%) (n = 102)	Caregivers number (%) (n = 102)	Total
*Age (year) (x̄± SD)*	69.50 ± 14.89	53.15 ± 12.95	61.33 ± 16.16
Female	42 (41.2)	80 (78.4)	122 (59.8)
Male	60 (58.8)	22 (21.6)	82 (40.2)
*Marital status*			
Married	82 (80.4)	94 (92.2)	176 (86.3)
Single	20 (19.6)	8 (7.8)	28 (12.7)
*Educational status*			
Primary school	76 (74.5)	67 (46.9)	143 (70.1)
Middle School	11 (10.8)	16 (15.7)	27 (13.2)
High School-University	15 (14.7)	19 (18.6)	34 (16.7)
*Employment status*			
Employed	12 (11.8)	16 (15.7)	28 (13.7
Unemployed	90 (88.2)	86 (84.3)	176 (86.3)
*Socio-economic level*			
Low	12 (11.8)	33 (32.4)	45 (22.1)
Middle	90 (88.2)	69 (67.6)	159 (77.9)
*Practicing religious ritual regularly*			
Yes	89 (87.3)	83 (81.4)	172 (84.3)
No	13 (12.7)	19 (18.6)	32 (15.7)
*Practicing religious ritual*			
Pray	73 (82.0)	55 (66.3)	128 (74.4)
Namaz*	7 (7.9)	19 (22.9)	26 (15.1)
Read the Qur’an	9 (10.1)	9 (10.8)	18 (10.5)
*Relationship to patients*			
Mother-father	–	40 (39.2)	40 (39.2)
Spouse only	–	34 (33.3)	34 (33.3)
Children only	–	5 (4.9)	5 (4.9)
Other	–	23 (11.3)	23 (11.3)
*Cancer type*			
Lung cancer	30 (14.7)	–	30 (14.7)
Head and neck cancer	17 (8.3)	–	17 (8.3)
Gastrointestinal cancer	16 (7.8)		16 (7.8)
Sarcoma	14 (6.9)		14 (6.9)
Gynecologically cancer	11 (5.4)	–	11 (5.4)
Breast cancer	8 (3.9)	–	8 (3.9)
Other	6 (2.9)		6 (2.9)
*Duration of cancer*			
6 mounth–1 year	60 (29.4)	–	
1–3 years	33 (16.2)	–	
> 3 yrs	9 (4.4)	–	

*Namaz: prayer performed by Muslims 5 times each day

The average total SCNI score was 71.00 ± 9.64 for the cancer patients and 64.94 ± 13.77 for their caregivers. The mean SCNI subscale scores of the cancer patients were 40.63 ± 7.61 for meaning and hope and 30.37 ± 3.56 for caring and respect, while these scores were 37.49 ± 9.54 and 27.45 ± 5.96, respectively, for the caregivers. The total SCNI and subscale scores of the patients and caregivers showed statistically significant differences (*p* < 0.05) (Table 2).

**Table 2:** Cancer patients and caregivers mean scores of the spiritual care needs inventory and its sub-dimensions (n = 204)

Inventory	Patients mean ± SD	Caregivers mean ± SD	Mean difference	t	df	*p*
Meaning and hope	40.63 ± 7.61	37.49 ± 9.54	3.14706	2.603*	192.568	0.010**
Caring and respect	30.37 ± 3.56	27.45 ± 5.96	2.92157	4.244	165.052	0.000
Total	71.00 ± 9.64	64.94 ± 13.77	6.068	3.645	180.810	0.000

Independent Sample t test *,* p* < 0.05**

Table 3 presents the correlation between the sociodemographic characteristics of the patients and their caregivers and their total SCNI scores. According to the statistical analysis, the SCNI scores of the patients did not significantly differ according to gender (t = − 1.158,* p* = 0.250), marital status (t = 0.407, *p*  = 0.685), educational level (F = 0.219,  *p*= 0.473), socio-economic status (t = − 0.526,* p* = 0.593), regular practise of religious rituals (t = − 0.333,* p* = 0.740), cancer type (F = 2.187,* p* = 0.051), or duration of cancer (F = 1.154,* p *= 0.320) (*p* > 0.05). However, employment status was significantly different for the SCNI scores of the patients, with the unemployed patients having a higher mean SCNI score than the employed patients (t = -3.219, *p = 0.004*).

**Table 3:** Socio-demographics and total spiritual care needs (*n* = 204)

Characteristics	Cancer patients		Caregivers	
	Mean ± SD	Test and* p* value	Mean ± SD	Test and* p* value
*Gender*				
Female	69.69 ± 9.11	t = − 1.158* *p* = 0.250	65.30 ± 14.20	t = 0.500* *p* = 0.618
Male	71.93 ± 9.96		63.63 ± 12.29	
*Marital status*				
Married	70.81 ± 10.24	t = 0.407 *p* = 0.685	65.00 ± 14.04	Z = − 1.185** *p* = 0.236
Single	71.80 ± 6.77		64.25 ± 10.79	
*Educational status*				
Primary school	71.25 ± 9.96	F = 1.595*** *p* = 0.208	63.82 ± 13.62	F = 1.703*** *p* = 0.187
Middle School	69.18 ± 4.11		63.43 ± 13.40	
High School-University	71.00 ± 11.10		70.15 ± 14.09	
*Employment status*				
Employed	66.49 ± 4.91	Z = − 2,157** *p = 0.03*	71.12 ± 11.71	Z = 2.196 *p* = 0.028
Unemployed	71.67 ± 9.93		63.79 ± 13.88	
*Socio-economic level*				
Low	72.41 ± 7.98	Z = − 0.832** p = 0. 406	67.69 ± 15.19	t = − 1.404 *p* = 0.163
Middle	70.82 ± 9.86		63.62 ± 16.9	
*Practicing religious ritual regularly*				
Yes	70.88 ± 10.01	Z = − 0.910** *p* = 0. 363	65.81 ± 13.89	t = -1.351* *p* = 0.180
No	71.84 ± 6.73		61.10 ± 12.89	
*Practicing religious ritual*				
Pray	70.42 ± 9.33	*X*^*2*^ = 0.315**** *p* = 0.854	66.01 ± 14.14	F = 0.947*** *p* = 0.392
Namaz	74.14 ± 15.63		67.89 ± 12.64	
Read the Qur’an	72.11 ± 11.11		60.22 ± 14.88	
*Relationship to patient*				
Mother-father	–	–	65.42 ± 14.64	F = 1.476*** *p* = 0.226
Spouse only	–		67.14 ± 14.47	
Children only	–		69.00 ± 9.48	
Other	–		59.95 ± 11.77	
*Cancer type*				
Lung cancer	74.06 ± 12.12	F = 1.160*** *p* = 0.367	–	–
Head and neck cancer	71.17 ± 5.38		–	
Gastrointestinal cancer	71.87 ± 11.50		–	
Sarcoma	67.00 ± 8.00		–	
Gynecologically cancer	68.36 ± 8.77		–	
Breast cancer	70.00 ± 6.8		–	
Other	68.50 ± 4.54			
*Duration of Cancer*				
6 mo–1 yr	70.16 ± 8.32	F = 1.154*** *p* = 0.320		
1–3 yrs	73.06 ± 12.01			
> 3 yrs	69.11 ± 7.65			–

Independent Sample t test*, Mann-Whitney U test **, One-Way ANOVA***, Kruskal Wallis test, **** p < 0.05

There was no statistically significant difference in the total SCNI scores of the caregivers according to gender (t = 0.500,* p*  = 0.618), marital status (t = − 0.147,* p* = 0.883), educational level (F = 2.175,* p* = 0.05), employment status (t = 1.984,  *p*= 0.0*5*), socio-economic level (t = − 1.404,* p* = 0.163), or regular practise of religious rituals (t = − 895,* p* = 0.061).

Pearson correlation coefficients were used to examine the correlation between the patients’ and their caregivers’ scores on the SCNI subscales. Moderately positive and significant (*p* < 0.05) correlations were found for the total SCNI scale(r = 0.449), the meaning and hope subscale (r = 0.378), and the caring and respect subscale (r = 0.546) (Table 4).

**Table 4:** Correlation between cancer patients and caregivers mean scores of the spiritual care needs inventory and its sub-dimensions

Caregivers (n = 102)			
*Patients (n = 102)*			
Sub-dimensions	Meaning and hope	Caring and respect	Total SCNI
Meaning and hope	r =378**	–	–
Caring and respect	–	r = 546**	–
Total SCNI	–	–	r = 449**

r = Pearson’correlation, *p*< 0·01

## Discussion

Spirituality is the universal and inner dimension of human beings, encompassing the significance, purpose, and principles of human existence, devoid of any religious affiliations. It is a personal journey pertaining to an individual’s quest for meaning in life and the establishment of connections with one’s own being, others, nature, and the divine deity (Balboni et al., 2022; Gijsberts et al., 2019). Patients with cancer may face challenges stemming from apprehension about the uncertain nature of their condition and anxiety about mortality, which are factors that have been associated with poor mental health outcomes (Paterson et al., 2022). Furthermore, caregivers encounter significant difficulties in providing care to patients, especially in cases where cancer is advanced or care is required over an extended period of time. During this process, they face emotional, financial, physical, spiritual, material, and existential challenges (O’Callaghan et al., 2020; Buck & McMillan, 2012). Nursing research should focus on caregivers’ spiritual needs, given the vital role that families play in promoting the overall well-being of patients (Taylor, 2003). The assessment of spiritual care needs is extremely important in meeting the needs of cancer patients (Bai & Lazenby, 2015; Yazgan & Demir, 2019). The objective of this study was to examine and compare the spiritual care needs of cancer patients receiving home care, as well as their caregivers.

There was no significant relationship between spiritual care needs and gender, marital status, educational level, or socio-economic status. The findings of this study exhibit slight variations when compared to those of previous studies. Riklikienė et al. (2020) and Nejat et al. (2023) found that the self-reported importance of spiritual needs was higher in women than in men. In another study, Cheng et al. (2018) concluded that the disease stage and hospitalization frequency of patients significantly affected their spiritual needs, whereas factors such as gender, marital status, and education level did not have a significant effect. These results can be explained by the variation in spiritual needs depending on cultural background.

In this study, the SCNI scores were found to be higher in unemployed patients compared to employed patients (*p *< 0.05). The literature indicates that there are significant variations in the domain of religious needs across different occupational statuses, and religious needs hold greater significance among housewives compared to individuals in other occupations (Nejat et al., 2023). Exposure to different values and worldviews may reduce the need for spiritual care.

This study revealed that patients with cancer had moderate spiritual care needs, which were at a higher level than those of their caregivers. Previous studies have also shown that cancer patients need more spiritual care than patients undergoing acute care (Wu et al., 2016), ostomy (Ayık et al., 2021), or orthopaedic surgery (Okgün Alcan et al., 2022). Patients with urinary incontinence have been shown to have even greater spiritual care needs (Özveren et al., 2022). The feasibility of making comparisons is hindered by the differences in the spiritual needs of different sample groups, which are affected by disease- and patient-related factors.

Patients with cancer require spiritual care, which encompasses being able to engage in religious rituals, discussing death, and establishing meaningful connections with others (Mesquita et al., 2017; Evans Webb et al., 2021). Cultural factors can also influence spiritual needs. A review found that individuals frequently expressed their spiritual needs related to maintaining a sense of tranquillity, cultivating optimism, and seeking and appreciating the meaning of life (Evans Webb et al., 2021). Although the provision of spiritual care is an important component in the overall treatment of cancer patients, there is evidence to suggest that these patients often perceive a lack of attention in this respect (Richardson, 2014). Healthcare professionals often overlook the spiritual needs of patients and their families due to insufficient skills, limited time availability, and a lack of confidence (Moore et al., 2013).

In the literature, praying or being prayed for is the most frequently reported religious need among patients (Evans Webb et al., 2021; Yazgan & Demir, 2019; Üstündağ & Zencirci, 2015; Dedeli et al., 2015). The current study did not examine the dimension of religious and cultural rituals in depth. Nevertheless, more than half of the participants stated they prayed as a form of spiritual practise. Cancer patients and their caregivers exhibited a heightened level of religious devotion when discussing their engagement in spiritual practises. Other spiritual practises included performing salah and reading the Quran. The sample mostly comprised Muslims and individuals with an Islamic background. These results suggest that the spiritual needs and mental state of patients may be affected by their religious beliefs and cultural background.

Strong spiritual beliefs often shape the experiences of caregivers providing care to patients with advanced cancer (Delgado-Guay et al., 2013). In this study, regular religious practices did not differ for the SCNI scores of the patients or their caregivers (*p* > 0.05). However, the literature presents different results and findings through the citations of diverse studies. For example, Wu et al. (2016) showed that acute care patients who engaged in regular religious practises had higher spiritual care needs. According to other studies, some patients experience a loss of faith as a result of their cancer diagnosis (Paterson et al., 2020), while some patients and their spouses find strength in nature and use faith and prayer as mechanisms for managing their conditions (Paterson et al., 2021). Caregivers have also been found to demonstrate an increased inclination towards praying and meditation subsequent to the diagnoses of their patients (O’Callaghan et al., 2020). To meet patient’s spiritual needs, it is necessary to perform a spiritual assessment based on their culture. Addressing the spiritual needs of cancer patients involves assessing their beliefs and relationships with family, friends, and the divine (Delgado-Guay, 2014; Maiko et al., 2019). There are studies in the literature assessing the spiritual care needs of Muslim cancer patients (Kırca et al., 2023; Dedeli et al., 2015). However, further research is warranted to examine the expectations of cancer patients regarding the spiritual support to be provided by nursing professionals.

### Study Limitations

The main limitation of this study is that it involved a small sample size from only one home care unit, which limits the correlations identified, thus generalizability is restricted and further research involving a larger sample size is warranted. Longitudinal studies on this subject are also recommended. Lastly, the provision of spiritual care can be influenced by various factors, such as attachment patterns, disease stage, caregiver traits, hospitalization frequency, and social support, which were not evaluated in the current study.

### Conclusion and Recommendations

According to the results of the study, cancer patients need more spiritual care than their caregiver. However, there is a positive correlation between the spiritual care needs of patients and their caregivers. Identifying the spiritual care needs of patients will help raise awareness of the importance of holistic care and improve the quality of care. Nurses, whose are primarily responsible for delivering care, play an important role in meeting the spiritual needs of cancer patients and their caregivers. The spiritual needs of cancer patients should be considered in care planning and provision processes. The spiritual needs of individuals exhibit variability, and the influence of religious practises on these needs manifests in a multitude of ways. To provide proper spiritual support, it’s crucial to consider individual beliefs and preferences. The interdisciplinary and culturally appropriate provision of spiritual care for cancer patients holds great importance. Primary home care health providers should incorporate spiritual care into their provision of services for cancer patients receiving treatment in a home setting. To reduce spiritual needs, doctors and nurses should frequently communicate with family caregivers. Caregivers of cancer patients should receive counselling, support, and spiritual care. Social services should support family caregivers who engage in extended durations of daily care giving.
